# Intra-S phase checkpoint kinase Chk1 dissociates replication proteins Treslin and TopBP1 through multiple mechanisms during replication stress

**DOI:** 10.1016/j.jbc.2022.101777

**Published:** 2022-02-26

**Authors:** Rebecca L. Kelly, Amelia M. Huehls, Annapoorna Venkatachalam, Catherine J. Huntoon, Yuichi J. Machida, Larry M. Karnitz

**Affiliations:** 1 Department of Molecular Pharmacology and Experimental Therapeutics, Mayo Clinic, Rochester, Minnesota, USA; 2 Division of Oncology Research, Mayo Clinic, Rochester, Minnesota, USA

**Keywords:** Treslin, TopBP1, Chk1, Chk1 inhibitors, cytarabine, replication stress, origin firing, AraC, cytarabine, CDK, cyclin-dependent kinase, Chk1, checkpoint kinase 1, RNL, arginine-asparagine-leucine, SFB, S-Tag, FLAG, streptavidin-binding peptide

## Abstract

Replication stress impedes DNA polymerase progression causing activation of the ataxia telangiectasia and Rad3-related signaling pathway, which promotes the intra-S phase checkpoint activity through phosphorylation of checkpoint kinase 1 (Chk1). Chk1 suppresses replication origin firing, in part, by disrupting the interaction between the preinitiation complex components Treslin and TopBP1, an interaction that is mediated by TopBP1 BRCT domain-binding to two cyclin-dependent kinase (CDK) phosphorylation sites, T968 and S1000, in Treslin. Two nonexclusive models for how Chk1 regulates the Treslin–TopBP1 interaction have been proposed in the literature: in one model, these proteins dissociate due to a Chk1-induced decrease in CDK activity that reduces phosphorylation of the Treslin sites that bind TopBP1 and in the second model, Chk1 directly phosphorylates Treslin, resulting in dissociation of TopBP1. However, these models have not been formally examined. We show here that Treslin T968 phosphorylation was decreased in a Chk1-dependent manner, while Treslin S1000 phosphorylation was unchanged, demonstrating that T968 and S1000 are differentially regulated. However, CDK2-mediated phosphorylation alone did not fully account for Chk1 regulation of the Treslin–TopBP1 interaction. We also identified additional Chk1 phosphorylation sites on Treslin that contributed to disruption of the Treslin–TopBP1 interaction, including S1114. Finally, we showed that both of the proposed mechanisms regulate origin firing in cancer cell line models undergoing replication stress, with the relative roles of each mechanism varying among cell lines. This study demonstrates that Chk1 regulates Treslin through multiple mechanisms to promote efficient dissociation of Treslin and TopBP1 and furthers our understanding of Treslin regulation during the intra-S phase checkpoint.

DNA replication requires the assembly of protein complexes on the DNA in a precisely regulated manner to promote efficient and error-free replication of the DNA (1). The formation of an active origin of replication involves the sequential events of origin licensing and origin firing. Origin licensing occurs in late M/early G1 phases of the cell cycle, during which inactive MCM2-7 complexes are loaded onto the DNA. During S phase, the licensed origins are converted to preinitiation complexes by the activities of DBF4-dependent kinase and cyclin-dependent kinase (CDK), which together regulate the assembly of rate-limiting factors to activate replicative helicases and fire origins (2, 3). One of the rate-limiting steps in this process is the phosphorylation of Treslin on threonine-968 (T968) and serine-1000 (S1000), two sites that conform to CDK2 consensus motifs and are CDK2 substrates *in vitro* (4, 5). These Treslin phosphorylations create binding sites for the N-terminal BRCT domains of TopBP1, thus driving the interaction of Treslin with TopBP1, an interaction that is required for loading the helicase component CDC45L onto the DNA and subsequent origin firing.

In response to replication stress, the firing of new origins is suppressed by the intra-S phase checkpoint (6, 7, 8). Replication stress in cancer cells has many sources, including endogenous DNA damage, difficult-to-replicate sections of DNA, oncogene-induced dysregulation of replication dynamics, chemotherapy-induced DNA damage, and antimetabolite-induced disruption of dNTP supply. Replication stress causes the uncoupling of the replicative helicase from DNA polymerase and results in stretches of single-stranded DNA (9, 10, 11). The formation of single-stranded DNA in turn leads to the activation of the ataxia telangiectasia and Rad3-related kinase signaling pathway, which promotes genome stability through the effector kinase checkpoint kinase 1 (Chk1) by arresting cell cycle progression, stabilizing replication forks, and suppressing new origin firing (6).

One of the mechanisms by which Chk1 negatively regulates origin firing is by disrupting the interaction between Treslin and TopBP1 (5). Two models have been proposed to explain how Chk1 inhibits this interaction (12, 13, 14, 15). In one model, Chk1 disrupts the Treslin–TopBP1 interaction by catalyzing phosphorylations that lead to the sequestration or degradation of CDC25 phosphatase family members that remove inhibitory phosphorylations on CDK2 (16). Decreased CDK2 activity would then lead to reduced phosphorylation of Treslin on the sites (T968 and S1000) required for its interaction with TopBP1. Notably, however, no studies have directly examined whether phosphorylation of these sites changes in response to replication stress. In the second model, Chk1 directly phosphorylates Treslin, thereby disrupting the Treslin–TopBP1 interaction in a process analogous to the mechanism by which the *S. cerevisiae* orthologs of Treslin and TopBP1 (Sld3 and Dpb11, respectively) are regulated by the replication stress–activated kinase Rad53 (17, 18). Here, we (i) directly examine whether Treslin T968 and S1000 phosphorylations change following replication stress and (ii) further show that both CDK2 and Chk1 phosphorylation sites on Treslin participate in Chk1 regulation of the Treslin–TopBP1 interaction and origin firing, with varying roles in different cell types.

## Results

### Chk1 regulates the Treslin–TopBP1 interaction

**Figure 1:**
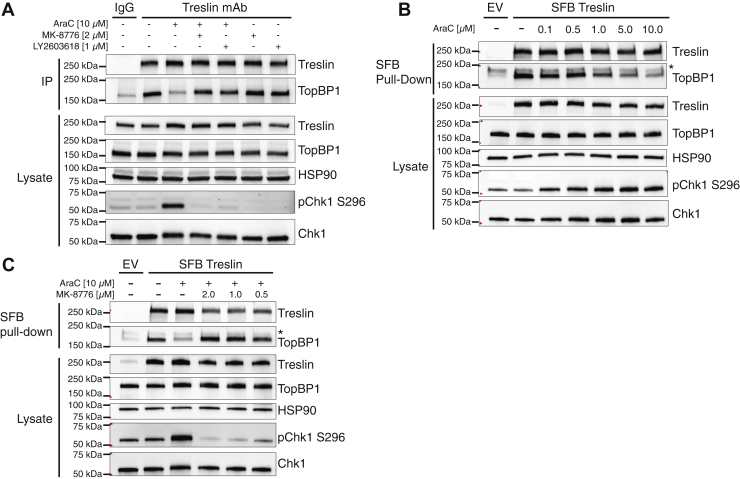
**Chk1 regulates the Treslin–TopBP1 interaction**. *A*, K562 cells were treated for 4 h with the indicated agents prior to lysis. Lysates were immunoprecipitated (IP) with mouse immunoglobulin G (IgG) control antibody or Treslin monoclonal antibody (mAb) prebound to protein G agarose beads. Immunoprecipitates and lysates were immunoblotted for the indicated antigens. *B*, K562 cells were transfected with empty vector (EV) or pIRES SFB Treslin WT, incubated for 24 h, treated with the indicated agents for 4 h, and lysed. Lysates were incubated with streptavidin-agarose beads for pulldown of SFB Treslin. Pulldowns and lysates were immunoblotted for the indicated antigens. *C*, as in B, but with indicated treatment conditions. ∗nonspecific band. AraC, cytarabine; Chk1, checkpoint kinase 1; SFB, S-Tag, FLAG, streptavidin-binding peptide.

To examine the role of Chk1 in replication stress-induced dissociation of Treslin and TopBP1, we first assessed the effects of the Chk1 inhibitor MK-8776 as well as LY2603618, a Chk1 inhibitor that was recently reported to be among the most specific kinase inhibitors known (19), on replication stress-induced dissociation of these proteins. Cytarabine (AraC), a nucleoside analog that blocks DNA replication following its incorporation by DNA polymerase, induced the dissociation of endogenous Treslin and TopBP1 (Fig. 1*A*), as did the replication inhibitors aphidicolin and hydroxyurea (Fig. S1). This dissociation was prevented by two different Chk1 inhibitors (Fig. 1*A*) as well as Chk1 siRNAs (Fig. S2). Consistent with these results, AraC induced dissociation of endogenous TopBP1 from transiently expressed SFB (S-Tag, FLAG, streptavidin-binding peptide)-tagged Treslin (SFB Treslin), and this dissociation was blocked by Chk1 inhibition (Fig. 1, *B* and *C*). Notably, the dissociation was inversely correlated with the degree of Chk1 activation as assessed by Chk1 autophosphorylation on serine-296 (S296).

### Chk1 activation decreases Treslin T968 but not S1000 phosphorylation

**Figure 2:**
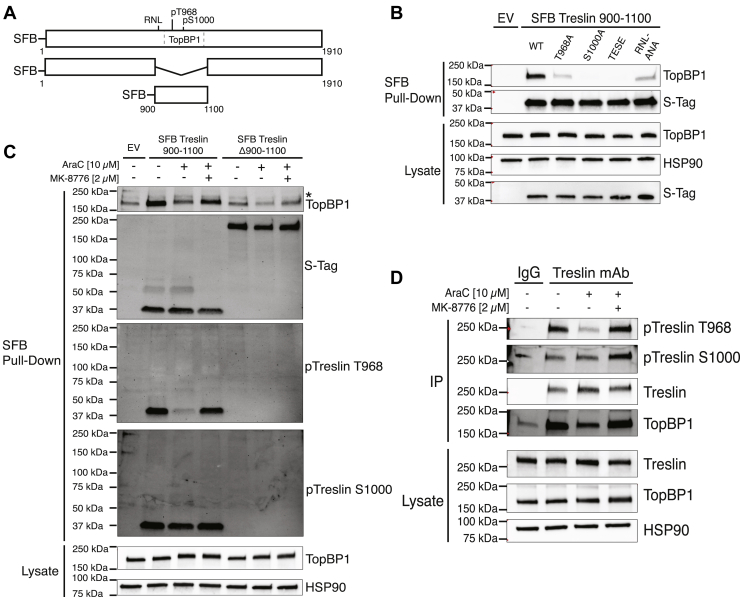
**Chk1 activation decreases Treslin T968 but not S1000 phosphorylation**. *A*, diagram of SFB Treslin (full-length Treslin, amino acids 1–1910), SFB Treslin Δ900–1100 (lacking amino acids 900–1100), and SFB Treslin 900–1100 (only amino acids 900–1100). Relative positions of the RNL sequence, T968 and S1000 sites, and TopBP1 binding region (*dashed rectangle*) are indicated on the full-length protein. *B*, K562 cells were transfected with WT pIRES SFB Treslin 900–1100 and indicated mutants, incubated for 24 h, and lysed. Lysates were incubated with streptavidin agarose beads to pulldown SFB Treslin fragments. Pulldowns and lysates were immunoblotted for the indicated antigens. *C*, K562 cells were transfected with pIRES SFB Treslin 900–1100 and pIRES SFB Treslin Δ900–1100, incubated for 24 h, treated with the indicated agents for 4 h, and lysed. Lysates were incubated with streptavidin agarose beads for pulldown of SFB Treslin fragments. Pulldowns and lysates were immunoblotted for the indicated antigens, including phospho-T968 Treslin (pTreslin T968) and phospho-S1000 Treslin (pTreslin S1000). *D*, K562 cells were treated for 4 h with the indicated agents prior to lysis. Lysates were incubated with protein G agarose beads prebound with a control mouse IgG monoclonal antibody or Treslin monoclonal antibody (mAb). Immunoprecipitates and lysates were immunoblotted for the indicated antigens. AraC, cytarabine; Chk1, checkpoint kinase 1; SFB, S-Tag, FLAG, streptavidin-binding peptide; RNL, arginine-asparagine-leucine.

For initial study of the interaction, we generated an expression vector for Treslin 900–1100 (Treslin amino acids 900–1100, Fig. 2*A*), which encompasses Treslin T968 and S1000, two CDK2 sites that are implicated in TopBP1 binding, and which also includes the RNL (arginine-asparagine-leucine) motif that targets CDK2 to phosphorylate Treslin (4). As shown in Figure 2*B*, SFB Treslin 900–1100 interacted with TopBP1, and conversion of either T968 or S1000 to alanine markedly reduced interaction with TopBP1, consistent with previous reports that these sites are required for stable interaction with TopBP1 (4, 5).

Based on a previous study showing that full-length Treslin TESE increased origin firing in cells expressing WT endogenous Treslin (20), we next assessed the effect of converting Treslin T968 and S1000 to glutamates in Treslin 900–1100 (Treslin 900–1100 TESE). Interestingly, Treslin 900–1100 TESE did not interact with TopBP1 (Fig. 2*B*), thus raising the possibility that these mutations do not augment origin firing by inducing constitutive interaction between Treslin and TopBP1. Finally, we examined how mutation of the Treslin RNL sequence, which is involved in recruiting CDK to its substrate (4), affected the interaction of Treslin with TopBP1. Treslin 900–1100 RNL-ANA also reduced interaction with TopBP1, confirming the importance of CDK recognition for interaction of Treslin 900–1100 with TopBP1 (Fig. 2*B*).

Because we observed that both Treslin T968 and S1000 are necessary for the TopBP1 interaction in cells, we developed phospho-specific antibodies to both sites (see Fig. S3, *A*–*D* for antibody validation). Using these antibodies, we next evaluated T968 and S1000 phosphorylation of Treslin 900–1100 in cells treated with AraC and MK-8776. As a negative control, we also included Treslin Δ900–1100, which lacks amino acids 900–1100 and therefore does not contain T968 and S1000 and should not interact with TopBP1. AraC decreased TopBP1 interaction with Treslin 900–1100 and reduced phosphorylation at Treslin T968 in a Chk1-dependent manner, while phosphorylation at S1000 was unaffected (Fig. 2*C*). As expected, Treslin Δ900 to 1100 did not react with either antiphospho-specific antibody and did not bind to TopBP1 above the background level seen in cells transfected with empty vector.

We then examined endogenous Treslin in K562 and U2OS cells treated with AraC and MK-8776 (Figs. 2*D* and S3*E*). As was observed with Treslin 900–1100, T968 phosphorylation was reduced by AraC treatment in a Chk1-dependent manner, whereas S1000 phosphorylation was not diminished by replication stress but did increase slightly with Chk1 inhibition. Taken together, these findings demonstrate that S1000 and T968 phosphorylation are differentially regulated by Chk1, with the Chk1-dependent decrease in T968 phosphorylation correlated with TopBP1 dissociation from Treslin. Additionally, the findings also suggest that one mechanism by which Chk1 regulates the interaction of Treslin with TopBP1 is by reducing phosphorylation of Treslin T968.

### Preserving Treslin T968 phosphorylation does not prevent the Treslin–TopBP1 dissociation

**Figure 3:**
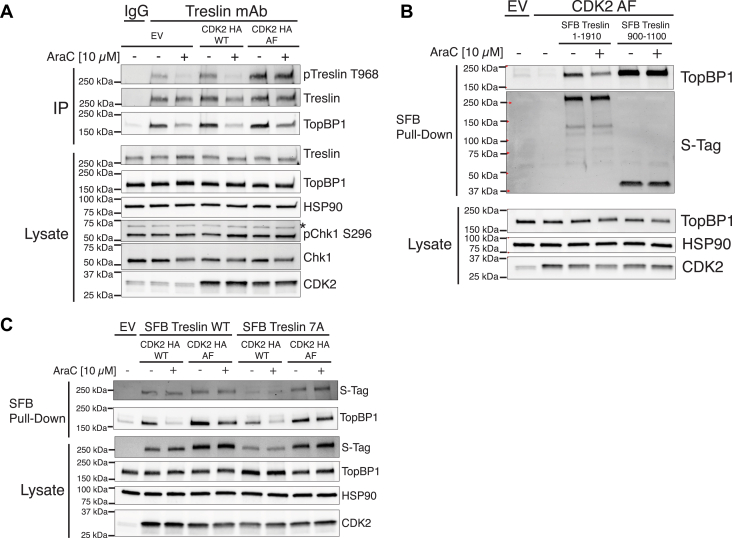
**Preserving Treslin T968 phosphorylation does not prevent the Treslin–TopBP1 dissociation**. *A*, K562 cells were co-transfected with empty vector (EV), pIRES SFB Treslin WT, or pIRES SFB Treslin 900–1100 with or without pCMV CDK2 HA AF (as indicated), incubated for 24 h, treated with 10 μM AraC for 4 h, and lysed. Lysates were incubated with streptavidin agarose beads to pulldown SFB-tagged proteins. Lysates and bead-bound proteins were immunoblotted for the indicated antigens. *B*, K562 cells were transfected with empty vector (EV), pCMV CDK2 HA WT, or pCMV CDK2 HA AF, incubated for 24 h, treated with 10 μM AraC for 4 h, and lysed. Lysates were incubated with protein G agarose beads prebound with mouse IgG control antibody or Treslin monoclonal antibody (mAb). Lysates and bead-bound proteins were immunoblotted for the indicated antigens. *C*, as in B, but K562 cells were transfected with empty vector (EV), pIRES SFB Treslin WT, or pIRES SFB Treslin 7A along with pCMV CDK2 HA WT or pCMV CDK2 HA AF, as indicated. CDK, cyclin-dependent kinase; AraC, cytarabine; Chk1, checkpoint kinase 1; SFB, S-Tag, FLAG, streptavidin-binding peptide.

To assess whether Chk1-mediated suppression of Treslin T968 phosphorylation fully accounts for the dissociation mechanism, we next sought a means to maintain T968 phosphorylation in the face of Chk1 activation. To that end, we expressed a constitutively active CDK2 in which threonine-14 and tyrosine-15 are converted to alanine and phenylalanine (CDK2 AF), respectively, thus uncoupling CDK2 from CDC25A regulation (21). Expression of Chk1-insensitive CDK2 AF, but not WT CDK2, enhanced basal T968 phosphorylation (Fig. 3*A*). Notably, Treslin T968 phosphorylation levels did not decrease in AraC-treated cells expressing CDK2 AF, thus demonstrating uncoupling of this phosphorylation site from Chk1 activation. Surprisingly, even though T968 phosphorylation was maintained during AraC treatment, AraC still induced Treslin to dissociate from TopBP1, suggesting that CDK2 regulation of Treslin T968 phosphorylation does not fully explain the Chk1-mediated dissociation of these proteins and that an additional Chk1-dependent mechanism regulates the Treslin–TopBP1 dissociation. Insight into this mechanism was provided when we used Treslin 900–1100. Unlike full-length Treslin, TopBP1 did not dissociate from Treslin 900–1100 when CDK2-AF was co-expressed in cells treated with AraC (Fig. 3*B*), indicating that a region(s) outside of Treslin 900–1100 is involved in the dissociation of Treslin from TopBP1.

### The Treslin C-terminal Chk1 binding domain regulates the Treslin–TopBP1 interaction by promoting Chk1 phosphorylation of Treslin near the TopBP1 binding region

Given that a previously identified C-terminal Chk1 binding site in Treslin was shown to regulate baseline origin firing (22), we hypothesized that this Chk1 binding site participated in regulating the Treslin–TopBP1 interaction in cells with Chk1-insensitive CDK2. To assess this possibility, we used a Treslin mutant (Treslin 7A, amino acids 1846–1852 converted to alanines) that does not bind Chk1 (22). Treslin 7A dissociated from TopBP1 in K562 cells treated with AraC (Fig. S4); however, co-expression of Treslin 7A with CDK2 AF reduced dissociation in cells treated with AraC (Fig. 3*C*), indicating that Treslin’s C-terminal Chk1 binding site participates in the dissociation.

**Figure 4:**
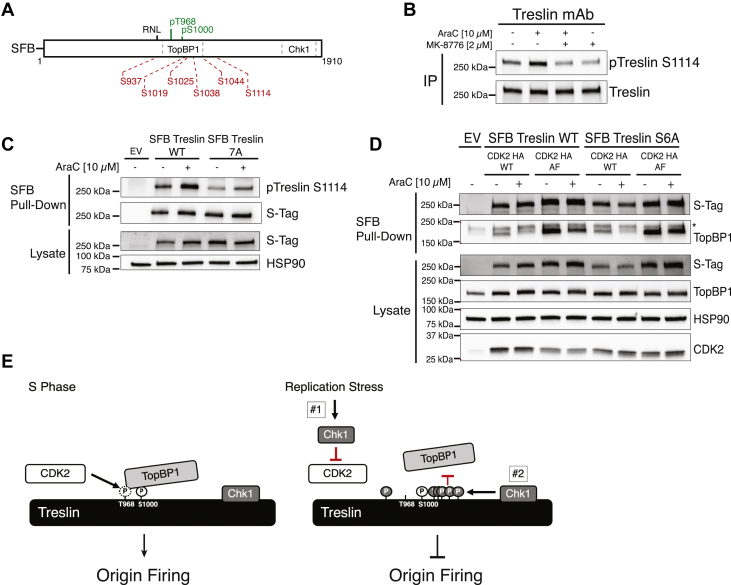
**The Treslin C-terminal Chk1 binding domain regulates the Treslin–TopBP1 interaction by promoting phosphorylation of Treslin near the TopBP1 binding region**. *A*, diagram depicting Treslin protein, T968 and S1000 phosphorylation sites (*green*), Chk1 phosphorylation consensus sites (*red*; S937, S1019, S1025, S1038, S1044, and S1114), and C-terminal Chk1 interaction region (*Chk1*). *B*, K562 cells were treated for 4 h with the indicated agents prior to lysis. Lysates were incubated with protein G agarose beads prebound with Treslin monoclonal antibody (mAb) for immunoprecipitation (IP). Bead-bound proteins were immunoblotted for phospho-S1114 Treslin (pTreslin S1114) and Treslin. *C*, K562 cells were transfected with empty vector (EV), pIRES SFB Treslin WT, or pIRES SFB Treslin 7A, incubated for 24 h, treated with 10 μM AraC for 4 h, and lysed. Lysates were incubated with streptavidin agarose to pulldown SFB-tagged proteins. Lysates and bead-bound proteins were immunoblotted for the indicated antigens. *D*, K562 cells were co-transfected with either empty vector, pIRES SFB Treslin WT, or pIRES SFB Treslin S6A along with pCMV CDK2 HA WT or pCMV CDK2 HA AF, as indicated, incubated for 24 h, treated for 4 h with 10 μM AraC, and lysed. Lysates were incubated with streptavidin agarose beads to pulldown SFB-tagged proteins. Lysates and bead-bound proteins were immunoblotted for the indicated antigens. ∗nonspecific band. *E*, Left panel: During origin firing, Treslin and TopBP1 interact through the N-terminal TopBP1 BRCT domains, which bind two phosphorylation sites on Treslin (T968 and S1000). Right panel: Replication stress activates Chk1, which has two mechanisms that influence the Treslin–TopBP1 interaction: #1 - Chk1 inhibits CDK activity through depletion and/or sequestration of CDC25 family phosphatases, #2 - Chk1 binds directly to the C terminus of Treslin and phosphorylates Chk1 sites on Treslin. Chk1 inhibitor blocks both mechanisms. AraC, cytarabine; Chk1, checkpoint kinase 1; SFB, S-Tag, FLAG, streptavidin-binding peptide; CDK, cyclin-dependent kinase.

Because Treslin 7A disrupted Chk1-induced dissociation of Treslin and TopBP1 in cells expressing CDK2 AF, we next explored the possibility that Chk1 phosphorylation of Treslin also regulates the Treslin–TopBP1 interaction. Interestingly, Treslin T968 and S1000 (shown in green) are flanked by multiple serine and residues (shown in red) that lie within Chk1 consensus sequences (R/K-X-X-S/T (23) (Figs. 4*A* and S5*A*), and phosphoproteomic analyses revealed that these sites are phosphorylated in cells (24). These observations raise the possibility (i) that these sites are phosphorylated by Chk1, (ii) that the C-terminal Treslin Chk1 binding site facilitates phosphorylation of these sites, and (iii) that they then contribute to the disruption of the Treslin and TopBP1 interaction. Consistent with this possibility, using a Treslin phospho-serine-1114 (S1114)–specific antibody (see Figs. S5, *B* and *C* and S3*D*) for antibody validation), we observed that AraC induced S1114 phosphorylation in a Chk1 activity–dependent manner (Fig. 4*B*) and that this phosphorylation was augmented by Treslin’s C-terminal Chk1 binding site (Fig. 4*C*).

To address whether Chk1 phosphorylation of Treslin in the vicinity of T968 and S1000 contributed to dissociation of Treslin from TopBP1, we mutated six serines (Fig. 4*A*, shown in red) embedded in Chk1 consensus sites to alanine to generate Treslin S6A. In contrast to WT Treslin, co-expression of CDK2 AF with Treslin S6A reduced the AraC-mediated dissociation of TopBP1 (Fig. 4*D*). Similar results were seen in HeLa cells (Fig. S6). Taken together, these results support a model in which Chk1 regulates the Treslin–TopBP1 interaction in K562 cells by two mechanisms: (#1) reducing T968 phosphorylation, a site required for TopBP1 binding, and (#2) phosphorylating Chk1 sites surrounding T968 and S1000 in a manner that is augmented by Chk1 binding to the C terminus of Treslin (Fig. 4*E*).

### Treslin S6A and CDK2 AF regulate DNA replication and origin firing during replication stress

**Figure 5:**
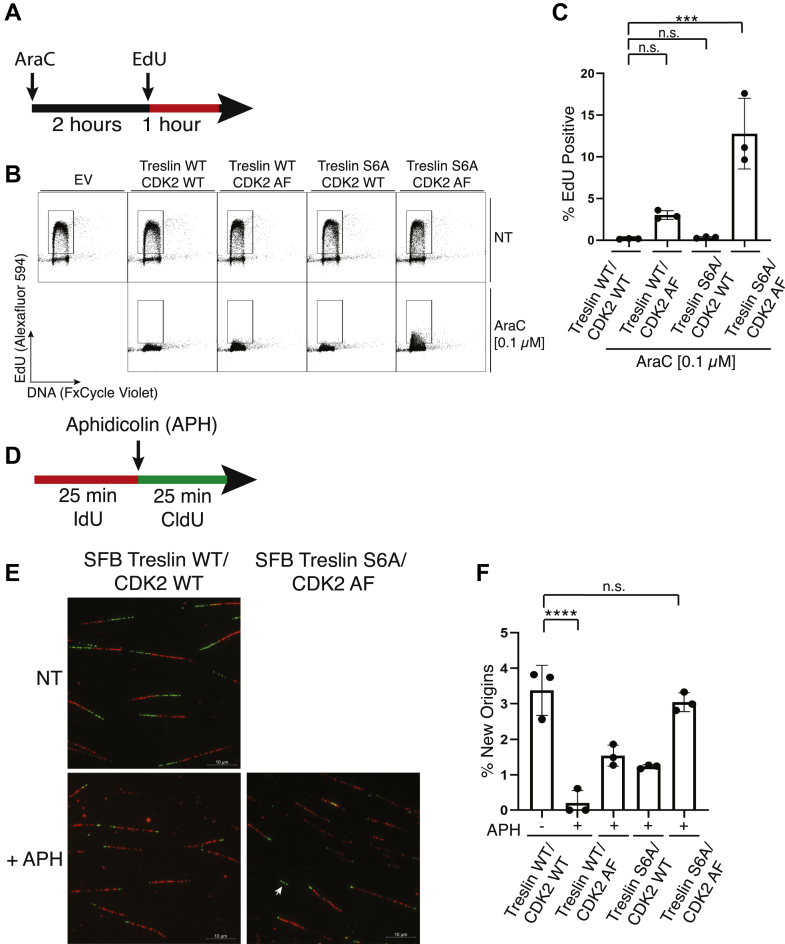
**Combined expression of Treslin S6A and CDK2 AF promote DNA synthesis and origin firing in AraC-treated K562 cells.***A*, schematic of EdU incorporation assay. Cells were treated with AraC for 3 h with EdU added during the last hour of treatment. *B*, scatter plots from EdU incorporation experiment. K562 cells were transfected with empty vector (EV) and indicated plasmids, cultured for 24 h, and treated with nothing (NT) or 0.1 μM AraC for 3 h. 10 μM EdU was added during the last hour of treatment. Samples were then collected, fixed, and stained with Alexafluor 594 for EdU and with FxCycle Violet for DNA content. *Gates* indicate EdU-positive cell population. *C*, bar graph shows the percentage of EdU-positive cells in AraC-treated conditions shown in *B*. Values are an average of three independent experiments with standard error (mean ± SEM). One-way ANOVA with Tukey’s multiple comparisons test was performed to assess the effects of SFB Treslin and CDK2 HA expression on EdU incorporation after AraC treatment (∗∗∗*p*-value < 0.0005). *D*, schematic of DNA fiber labeling. K562 cells were transfected with indicated plasmids, cultured for 24 h, pulse labeled with IdU (*red*), washed, treated with nothing or 0.5 μM aphidicolin (APH), and pulsed with CldU (*green*). *E*, representative images are shown for WT/WT, NT, WT/WT +APH, and S6A/AF, +APH. A *white arrow* is shown indicating a new origin. *F*, bar graph shows the percentage of new origins *versus* total events counted in each condition in E. At least 300 events were counted for each condition. Values are an average of three independent experiments with standard error (Mean ± SEM). Two-way ANOVA with Tukey’s multiple comparisons test was performed to assess the effects of SFB Treslin and CDK2 HA expression on origin firing. (∗∗∗∗*p*-value < 0.0001). AraC, cytarabine; Chk1, checkpoint kinase 1; SFB, S-Tag, FLAG, streptavidin-binding peptide; CDK, cyclin-dependent kinase.

Up to this point, our results show that in K562 cells, replication stress disrupts the Treslin–TopBP1 interaction by (i) reducing Treslin T968 phosphorylation levels and (ii) activating Chk1-mediated Treslin phosphorylation on sites in the vicinity of the Treslin–TopBP1 interaction. Given that the Treslin–TopBP1 interaction is required for origin firing, we reasoned that CDK2 AF and Treslin S6A may dysregulate DNA synthesis in AraC-treated K562 cells. In cells separately expressing CDK2 AF or Treslin S6A, a low concentration of AraC resulted in near-complete suppression of EdU incorporation (Fig. 5, *A* and *B*), and this incorporation did not differ significantly from cells expressing WT Treslin and CDK2 WT (Fig. 5*C*). In contrast, co-expression of Treslin S6A and CDK2 AF increased EdU incorporation in the presence of AraC, a result that is consistent with our observation that co-expression of Treslin S6A and CDK2 AF prevented AraC-induced dissociation of Treslin and TopBP1.

We next asked if Treslin S6A and CDK2 AF also affected origin firing using DNA fiber labeling to identify new origin firing (Fig. 5*D*). For these studies, we used aphidicolin because it rapidly inhibits DNA replication, unlike AraC, which needs to be metabolically activated and is therefore not amenable to DNA fiber labeling for new origins. Consistent with the observation that co-expression of Treslin S6A and CDK2 AF prevented replication stress–induced dissociation of Treslin and TopBP1, Treslin S6A and CDK2 AF co-expression also promoted origin firing during aphidicolin-induced replication stress (Fig. 5, *E* and *F*).

**Figure 6:**
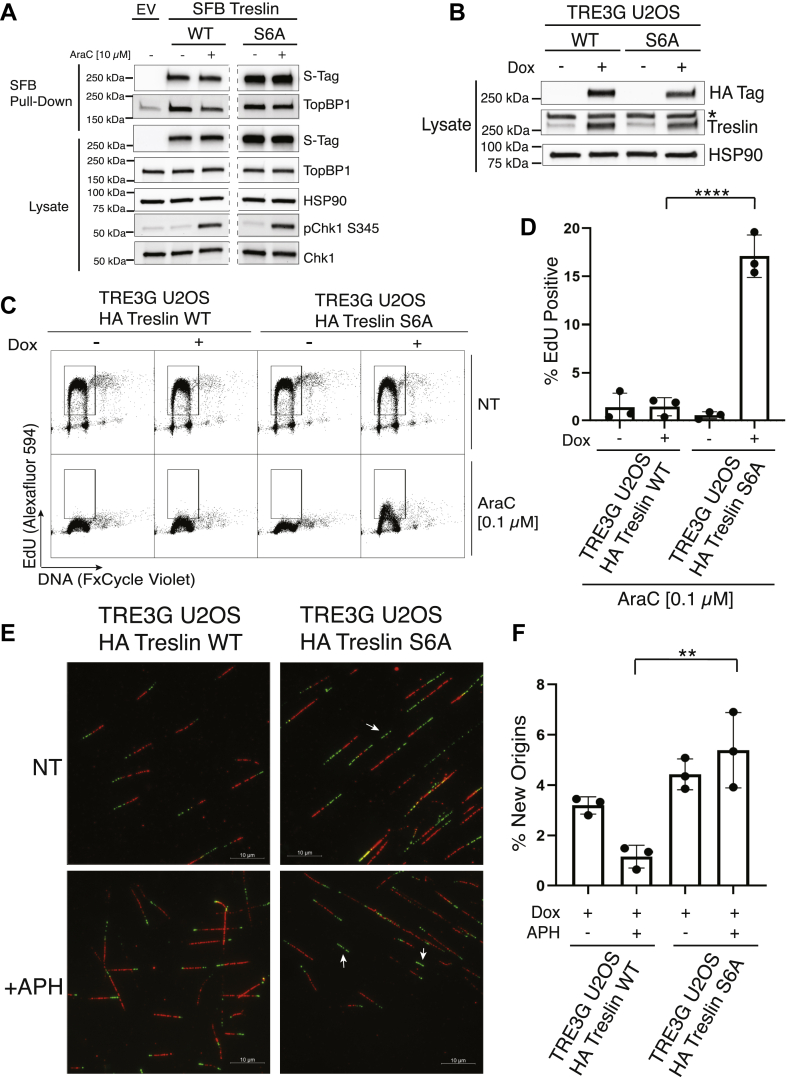
**Treslin S6A alone promotes DNA synthesis and origin firing in U2OS cells.***A*, U2OS cells were transfected with empty vector (EV), SFB-tagged wildtype Treslin (WT), or Treslin S6A (S6A), cultured for 48 h, treated with nothing (-) or 10 μM AraC for 2 h, and lysed. Cell lysates were incubated with streptavidin agarose beads to pulldown SFB-tagged proteins. Pulldowns were run on a gel and immunoblotted for the indicated antigens. All samples were run on the same gel, and extraneous lanes were cropped out of the immunoblot images and are indicated by dashes. *B*, TRE3G U2OS cell lines transduced with HA-tagged wildtype Treslin (WT) or HA-tagged Treslin S6A (S6A) were treated for 24 h with nothing (−) or 10 ng/ml (+) doxycycline (Dox) to induce HA Treslin expression. Cell lysates were immunoblotted for the indicated antigens. ∗nonspecific band. *C*, EdU incorporation assay as in schematic Figure. 5*A* was performed with TRE3G U2OS cell lines. U2OS cells were treated with 10 ng/ml doxycycline, cultured for 24 h, and treated with nothing (no treatment, NT) or 0.1 μM AraC for 3 h. 10 μM EdU was added during the last hour of treatment. Samples were then collected, fixed, and stained for EdU incorporation (Alexafluor 594) and DNA content (FxCycle Violet). *D*, bar graph shows the percentage of EdU-positive cells in each condition from C. Values are an average of three independent experiments with standard error (mean ± SEM). One-way ANOVA with Tukey’s multiple comparisons test was performed to assess the effects of HA Treslin on EdU incorporation after AraC treatment (∗∗∗∗*p*-value < 0.0001). *E*, DNA fiber labeling was performed as in the schematic in Figure 5*D*. U2OS cells were treated with 10 ng/ml doxycycline 24 h prior to assay. Cells were pulse labeled with IdU (*red*), washed, treated with nothing or 5 μM aphidicolin (APH), and pulsed with CldU (*green*). Representative images of the data are shown. *White arrows* indicate new origins. *F*, bar graph shows the percentage of new origins in each condition from E. Values are an average of three independent experiments with standard error (mean ± SEM). Two-way ANOVA with Tukey’s multiple comparisons test was performed to assess the effects of HA Treslin expression on origin firing. (∗∗*p*-value < 0.005). AraC, cytarabine; Chk1, checkpoint kinase 1; SFB, S-Tag, FLAG, streptavidin-binding peptide.

To assess the impact of Treslin S6A on TopBP1 dissociation and DNA replication in another cell line, we expressed WT and mutant Treslin in U2OS cells. In contrast to what we observed in K562 cells, expression of Treslin S6A alone affected TopBP1 dissociation and DNA replication. As shown in Figure 6*A*, AraC-induced TopBP1 dissociation was impaired in U2OS cells expressing Treslin S6A, suggesting that Treslin’s Chk1 phosphorylation sites play a more prominent role in regulating the Treslin–TopBP1 interaction in U2OS cells compared to K562 cells. To investigate how Treslin S6A affects DNA synthesis in the face of replication stress in U2OS cells, we developed cells that express WT Treslin or Treslin S6A in a doxycycline-dependent manner (Fig. 6*B*). Consistent with the fact that Treslin S6A had a TopBP1 dissociation defect in response to AraC (Fig. 6*A*), more EdU was incorporated in cells expressing Treslin S6A than in cells expressing WT Treslin after AraC treatment (Fig. 6, *C* and *D*). Aphidicolin did not suppress new origin firing in U2OS cells expressing Treslin S6A compared to cells expressing WT Treslin (Fig. 6, *E* and *F*).

## Discussion

Chk1 activation disrupts the interaction of Treslin with TopBP1, and this disruption is one mechanism by which Chk1 promotes the S-phase checkpoint and suppresses late-origin firing. However, the mechanism by which Chk1 disrupts the Treslin–TopBP1 interaction has remained obscure. On one hand, it has been proposed that Chk1 reduces phosphorylation of T968 and S1000, two CDK2 consensus sites, by attenuating the activity of CDC25 family members that activate CDKs, which would reduce TopBP1 binding to Treslin. On the other hand, Chk1 could directly phosphorylate Treslin and thereby disrupt the Treslin–TopBP1 interaction as has been observed for the *S. cerevisiae* orthologs of Treslin and TopBP1. To gain insight into how Chk1 disrupts the Treslin–TopBP1 interaction, we undertook a comprehensive analysis of the dissociation of the proteins in cancer cell line models.

Using Chk1 inhibitors and Chk1 siRNAs, we confirmed that Chk1 regulates the Treslin–TopBP1 interaction (Fig. 1), and our experiments confirmed the importance of both Treslin T968 and S1000 for interaction of Treslin and TopBP1. To study regulation of these sites, we developed phospho-specific antibodies for each site. To our knowledge, this study is the first to directly assess how replication stress affects the phosphorylation of T968 and S1000 using phospho-specific antibodies rather than TopBP1 binding as a proxy for Treslin phosphorylation on these sites (25). Our studies revealed that Treslin T968 phosphorylation is reduced in AraC-treated cells, consistent with Chk1-induced downregulation of CDK activity, the kinase thought to phosphorylate this site (Fig. 2). In contrast, S1000 phosphorylation was not concomitantly reduced by AraC but was modestly increased by Chk1 inhibition, suggesting that S1000 is differentially regulated compared to T968. Several potential mechanisms could explain this differential regulation. For example, S1000 may not be phosphorylated by CDK but instead be phosphorylated by another kinase that is not regulated by Chk1. Alternatively, S1000 may be a CDK substrate that is more effectively phosphorylated by CDK such that a reduction in CDK activity does not lead to substantial changes in S1000 phosphorylation. Finally, S1000 may be dephosphorylated much more slowly than T968, possibly because T968 is more accessible to phosphatases or because S1000 is protected from phosphatase activity. Consistent with these possibilities, a recent study found that Treslin S1000 could be subject to unique modulation by CDK-adaptor proteins CKS1 and CKS2 (25).

Having observed that Chk1 activation reduced phosphorylation of Treslin T968, we blocked Chk1-regulated loss of Treslin T968 phosphorylation by expression of CDK2 AF, whose activity is not suppressed by Chk1. Surprisingly, even when Treslin T968 phosphorylation was maintained by CDK2 AF in K562 cells, AraC still caused Treslin to dissociate from TopBP1, indicating that an additional mechanism plays a role in the dissociation. In an observation reminiscent of the Rad53-based regulation of the interaction of the Treslin and TopBP1 orthologs (Sld3 and Dpb11, respectively) in *S. cerevisiae*, we found that Treslin was directly phosphorylated on S1114 by Chk1 in response to replication stress and that Chk1 phosphorylation of S1114 (and other Chk1 sites) participated in Treslin–TopBP1 dissociation. Interestingly, Treslin S1114 phosphorylation was augmented by a Chk1 binding region in the C terminus of Treslin that was previously shown to suppress origin firing *via* an uncharacterized mechanism (22), suggesting that this Treslin Chk1 binding site regulates origin firing, at least in part, by enhancing phosphorylation of Treslin.

Additional studies then showed that in K562 and HeLa cells, both mechanisms had to be disabled by co-expression of Treslin S6A and CDK2 AF to block AraC-induced dissociation of Treslin and TopBP1 (Fig. 4). Consistent with this observation, co-expression of both Treslin S6A and CDK2 AF increased the level of DNA synthesis and origin firing in the face of replication stress in K562 cells, indicating that both mechanisms play a role the replication stress-induced S-phase checkpoint. In contrast, in U2OS cells, expression of Treslin S6A alone reduced the dissociation of Treslin and TopBP1 in response to replication stress. Correspondingly, in U2OS cells exposed to replication stress, Treslin S6A alone promoted EdU incorporation and origin firing, suggesting that the relative importance of each mechanism may differ among cell lines.

Our studies raise the question of how Treslin is differentially regulated in these two cell lines. One possibility is that CDK2 may be hyperactive in U2OS cells and therefore does not require the expression of constitutively active CDK2 to promote firing. This would be consistent with the observation that U2OS cells are especially sensitive to Chk1 inhibition (26, 27). Alternatively, additional signaling pathways may regulate the Treslin–TopBP1 interaction. On the one hand, unknown posttranslational modifications of Treslin may impact Treslin subcellular localization, conformation, or interactions with other proteins that could alter its interaction with TopBP1. On the other hand, signaling pathways that impinge on TopBP1 could also affect the relative importance of CDK2 *versus* Chk1. For example, the acetylation of several TopBP1 lysines is regulated by a SIRT1-dependent metabolic pathway that alters TopBP1 structure and its interaction with Treslin (28), raising the possibility that differences in metabolism could affect the differing requirements for CDK2 *versus* Chk1 phosphorylation of Treslin and its interaction with TopBP1. Additionally, AKT phosphorylation of TopBP1 promotes its interaction with EF21 and reduces its interaction with Treslin, suggesting that this signaling pathway could also lead to differences in the relative roles of CDK2 and Chk1 in regulating the Treslin–TopBP1 interaction (29). Accordingly, additional studies will be required to determine the complex, interacting regulatory mechanisms that control a cell’s critical decision to fire origins of replication.

In summary, this study of Treslin regulation during the replication stress checkpoint identified two mechanisms by which Chk1 signaling modifies Treslin to influence TopBP1 binding and origin firing, and they support a model in which Chk1 regulates the Treslin–TopBP1 interaction by reducing CDK2 activity, leading to reduced T968 phosphorylation and by phosphorylating Treslin S1114 and other Treslin Chk1 sites.

## Experimental procedures

### Cell culture

K562, U2OS, and HeLa cells were obtained from ATCC, cultured in RPMI 1640 (Corning) supplemented with 8% fetal bovine serum, and reinitiated from early-passage stocks every 3 months. Cells were confirmed negative for *mycoplasma* contamination with MycoAlert *Mycoplasma* Detection Kit (LT07–218, Lonza).

### Compounds

Cytarabine (catalog no. C6645) and hydroxyurea (catalog no. H8627) were from Sigma-Aldrich Co. MK-8776 (catalog no. S2735), and LY2603618 (catalog no. S2626) was obtained from Selleck Chemicals. Aphidicolin (catalog no. 32774) was obtained from Cell Signaling Technology, Inc. Doxycycline was from Clontech Labs (catalog no. 631311).

### Plasmid transfection

K562 cells (1 ×10^7^ cells/transfection) were electroporated with plasmid DNA using two 10-ms, 280-V pulses with an ECM 830 Square Wave (BTX) electroporator. U2OS and HeLa cell lines were transfected *via* forward-transfection with Lipofectamine 3000 (Invitrogen, catalog no. L3000015) following the manufacturer’s protocol.

### Plasmids and cloning

To construct expression vectors for full-length Treslin, Treslin Δ900–1100, and Treslin 900–1100, PCR amplification products were cloned into pIRES2-EGFP (Clontech) bearing an N-terminal SFB tag. Mutations were introduced using PCR amplification with mismatched primers. Plasmid pCMV Cdk2 HA (Addgene plasmid #1884; http://n2t.net/addgene:1884; RRID:Addgene_1884) was a gift from Sander van den Heuvel. pCMV CDK2 HA AF mutant (T14A/Y15F) was produced from plasmid pCMV Cdk2 HA using mismatched primer PCR amplification. TRE3G HA Treslin plasmids were generated by PCR amplification of Treslin from pIRES2-EGFP vectors and ligation into the new vector after restriction enzyme digest.

### Lysis and immunoprecipitation

Cells were lysed in a high salt buffer (20 mM Tris, 5 mM MgCl_2_, 500 mM NaCl, 0.5% Triton X-100 supplemented with 1 μg/ml pepstatin, 20 ng/ml microcystin, 10 μg/ml aprotinin, 20 μg/ml leupeptin, 30 μg/ml glycerol 2-phosphate, and 2 μg/ml sodium orthovanadate) for 5 min on ice and diluted with supplemented lysis buffer lacking NaCl to a final concentration of 150 mM NaCl. Pierce Universal Nuclease (Thermo Fisher Scientific, catalog no. 88702) was added to the lysate, and lysates were incubated on ice for 30 min prior to centrifugation at 16,000*g* for 5 min. Supernatants were then used for pulldown, immunoprecipitation, or immunoblotting as specified.

For streptavidin pulldown and immunoprecipitation experiments, cell lysates were incubated with streptavidin-coated beads or protein G beads prebound with antibody, respectively, at 4 °C for 1 h with rotation. Beads were then washed three times in lysis buffer, and proteins were eluted with 2× Laemmli sample by heating to 95 °C for 10 min.

### Immunoblotting

Proteins were separated by SDS–polyacrylamide gel electrophoresis on 4 to 15% TGX gels (BioRad, catalog no. #4561086) and transferred to polyvinyl difluoride membrane (Millipore, catalog no. IPVH00010). Membranes were blocked in 5% nonfat dried milk in Tris-buffered saline containing 0.1% Tween 20 (TBST), incubated overnight with primary antibody, washed three times with TBST, incubated with horseradish peroxidase-conjugated secondary antibody for 1 h, washed three times with TBST, incubated with SuperSignal West Pico PLUS Chemiluminescent Substrate (Thermo Fisher Scientific, catalog no. 34579), and imaged using a ChemiDoc MP imager with Image Lab software (BioRad). Primary antibodies used in this study are indicated in Table S1. Secondary antibodies used in this study are indicated in Table S2.

### Treslin mouse antibody

A mouse monoclonal antibody was generated by immunization with GST-Treslin (amino acids 882–1257) and counter-screening against GST by the Mayo Clinic Monoclonal Antibody Core facility.

### Treslin rabbit antibodies

Phosphorylated peptides corresponding to the indicated Treslin amino acids (Table S3) were synthesized and conjugated to keyhole limpet hemocyanin by the Mayo Clinic Medical Genome Facility Proteomics Core. Rabbits were immunized with keyhole limpet hemocyanin-conjugated peptides, and rabbit antisera were prepared by Cocalico Biologicals Inc. The antibody for total Treslin was purified using peptide conjugated to SulfoLink Coupling Resin (Thermo Fisher Scientific, catalog no. 20401) as described (30). Phospho-specific antibodies were purified from antisera by first removing nonphospho-specific antibodies using nonphosphorylated peptide coupled to SulfoLink Coupling Resin followed by binding to and elution from phosphopeptide-conjugated resin.

### siRNA transfection

U2OS cells (4 ×10^6^/transfection) cells were electroporated at 225 V with one 20-msec pulse with 20 μl of 20 μM siRNA in 200 μl total volume and plated in 10-cm dishes.

siRNAs used: control Luciferase: CUUACGCUGAGUACU UCGA, Chk1 #1: AAAGAUAGAUGGUACAACA, Chk1#2: GCAACAGUAUUUCGGUAUA

### Lambda phosphatase assay

After SFB Treslin pulldown, as described above, beads were washed twice with phosphatase buffer (50 mM Hepes, pH 7.5, 100 mM NaCl, 2 mM DTT, 0.01% Triton X-100) and resuspended in a final reaction volume of 50 μl in phosphatase buffer supplemented with 1 mM MnCl_2_. Lambda protein phosphatase (1 μl, New England BioLabs Inc, catalog no. P0753S) was added to the indicated samples, and samples were incubated for 30 min in a 30 °C water bath. The reaction was stopped by washing beads with lysis buffer.

### Doxycycline-inducible HA Treslin expression system in U2OS cell line

For TRE3G U2OS HA Treslin cell lines, the Lenti-X Tet-On 3G Inducible Expression System (Clontech Laboratories, Inc, catalog no. 631187) was used according to manufacturer’s protocol (31). In short, U2OS cells were transduced with Tet-On 3G and TRE3G HA Treslin lentiviruses, and enriched populations were selected with puromycin (2 μg/ml) and G418 (600 μg/ml). Cell lines were cultured in puromycin and G418 throughout the experiments. HA Treslin was induced with 10 ng/ml doxycycline 24 h prior to assay unless otherwise stated. Expression of HA-tagged protein was confirmed by Western blot.

### DNA fiber labeling

We followed the method described by Quinet *et al* (32) with some changes. In brief, transfected K562 or TRE3G cell lines were pulse labeled with 25 μM IdU for 25 min, washed with PBS (37 °C), refed with media (37 °C) containing 250 μM CldU with or without aphidicolin, and cultured for 25 min. Cells were then washed in PBS and diluted with ice-cold PBS. Cells (∼100,000) were lysed in 15 μl fiber lysis buffer (200 mM Tris-HCl, pH 7.4, 50 mM EDTA, and 0.5% SDS) on a microscope slide, and the slide was tilted to a 25° angle to allow fibers to spread along the length of the slide. Fibers were fixed in 3:1 methanol/acetic acid for 10 min, washed twice with PBS, and denatured for 80 min in 2.5 M hydrochloric acid. Slides were washed twice with PBS, blocked in 5% bovine serum albumin in PBS for 30 min, and stained with primary antibodies overnight (1:25 mouse anti-BRDU (BD Biosciences, catalog no. 347580), 1:1000 Rat anti-BRDU (Abcam, plc, catalog no. ab6326) diluted in 5% BSA in PBS). The next morning, slides were washed twice with PBS, stained with secondary antibodies [1:500 Alexafluor 594 anti-mouse (Invitrogen, catalog no. A11032) and 1:500 Alexafluor 488 anti-rat (Invitrogen, catalog no. A110066)] for 2 h and washed three times with PBS. SlowFade Gold 30 (μL) (ThermoFisher, catalog no. S36936) was added, and coverslips were applied. Images of fibers were acquired at 63× magnification on an Axio Observer seven scanning microscope (Ziess). Replication events were classified as ongoing fork, red-green; initiation, green-red-green; termination, red-only and red-green-red; and new origin, green-only (Fig. S7). More than 300 events were counted for each condition. Results are an average of three independent experiments.

### EdU incorporation

For EdU incorporation assays, the Click-iT Plus EdU Alexa Fluor 594 Flow Cytometry Assay Kit (ThermoFisher, catalog no. C10646) was used according to manufacturer’s protocol. In short, transfected K562s or TRE3G U2OS cells were treated with AraC for 3 h with 10 μM EdU added during the last hour. Cells were collected, and soluble proteins were extracted with a potassium phosphate buffer (10 mM potassium phosphate, pH 7.4, 0.1% Ipgal-CA-630, 10 mM NaCl, 5 mM MgCl_2_) as described (33). Cells were fixed by addition of 250 μl of potassium phosphate buffer containing 10% paraformaldehyde to give a final concentration of 2.5% paraformaldehyde for 1 h on ice and washed with ice-cold PBS. Cells (1 ×10^6^/sample) were stained for EdU with ClickIT Alexafluor 594 (ThermoFisher, catalog no. C10330) and DNA with FxCycle Violet (ThermoFisher, catalog no. F10347), according to manufacturer’s protocol. Cells were analyzed on a FACSCanto X SORP (BD Biosciences). Data were analyzed with FlowJo 10.6.1 (©Becton Dickinson and Company). EdU positive cells were gated to determine the EdU-positive population.

### Statistical analysis

Data were analyzed for statistical significance using GraphPad Prism software to perform analysis of variance (one-way or two-way as appropriate) with post-hoc multiple comparisons. We define a *p*-value of less than 0.05 as significant. Results are the mean and standard error from three independent experiments, and specific tests used are described in the figure legend.

## Data availability

All relevant data are contained within this manuscript.

## Supporting information

This article contains supporting information (34).

## Conflict of interest

The authors declare that they have no conflicts of interest with the contents of this article.
