# Supplementary material for: Intra-S phase checkpoint kinase Chk1 dissociates replication proteins Treslin and TopBP1 through multiple mechanisms during replication stress
Source: J Biol Chem. 2022 Feb 26;298(4):101777. doi: 10.1016/j.jbc.2022.101777 (PMC8965152; doi:10.1016/j.jbc.2022.101777)
Supplement: Supplemental Tables S1–S3 and Figures S1–S7 [file mmc1.pdf]

Intra-S phase checkpoint kinase Chk1 dissociates replication proteins Treslin and TopBP1 through multiple mechanisms during replication stress

Rebecca L. Kelly, Amelia M. Huehls, Annapoorna Venkatachalam, Catherine J. Huntoon, Yuichi J. Machida, and Larry M. Karnitz

Supporting Information:

Supporting Tables S1 - S3

Supporting Figures S1 – S7

Supporting Table S1 - Primary antibodies used in this study

Antibody	Species	Company	Dilution
CDK2	Rabbit mAb	ab32147, Abcam	1:1000
pChk1 S296	Rabbit mAb	D3O9F, Cell Signaling Technology, Inc.	1:1000
pChk1 S345	Rabbit mAb	133D3, Cell Signaling Technology, Inc.	1:1000
Chk1	Mouse mAb	sc-8408, Santa Cruz Biotechnology, Inc.	1:1000
FLAG (DYKDDDDK Tag)	Rabbit mAb	D6W5B, Cell Signaling Technology, Inc.	1:1000
HSP90	Mouse mAb	H9010, D. Toft, Mayo Clinic	1:20,000
Mouse IgG	Mouse	0107-01, Southern Biotech	IP - 1:50
TopBP1	Rabbit pAb	A300-111A, Bethyl Laboratories, Inc.	1:500
Treslin	Rabbit pAb	this publication	1:1000
Treslin	Mouse mAb	this publication	IP - 1:50
Treslin	Rabbit pAb	A303-472A, Bethyl Laboratories, Inc.	1:1000
S-Tag	Mouse mAb	Hackbarth et al.(1)	1:10,000

Supporting Table S2 - Secondary antibodies used in this study

Antibody	Company	Dilution
Anti-rabbit IgG HRP-linked	7074S, Cell Signaling Technology, Inc.	1:5000
Anti-mouse IgG HRP-linked	7076S, Cell Signaling Technology, Inc.	1:5000

Supporting Table S3 – Peptides for Treslin rabbit antibody production

Antibody	Synthetic peptide	Treslin amino acids
Total Treslin	SVSQPKSRSVQRVHSFQ - C	1030-1046
pTreslin T968	LLTKSVAE(p)TPVHKQIS -C	960-975
pTreslin S1000	DIGVVEE(p)SPEKGDEI -C	993-1007
pTreslin S1114	KKSHQKSL(p)SFSKTTPRR - C	1106-1122

Supporting Figure S1

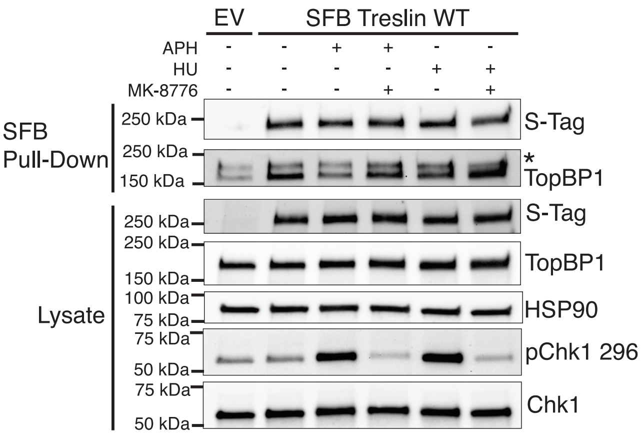

Supporting Figure S1. K562 cells were transfected with either empty vector (EV) or pIRES SFB Treslin WT, incubated for 24 hours, treated with either no treatment or 5 µM Aphidicolin (APH), 10 µM hydroxyurea (HU) with or without 2 µM MK-8776 for 4 hours, and lysed. Lysates were incubated with streptavidin agarose beads for pulldown of SFB Treslin. Pulldowns and lysates were immunoblotted for the indicated antigens. *, nonspecific band.

Supporting Figure S2

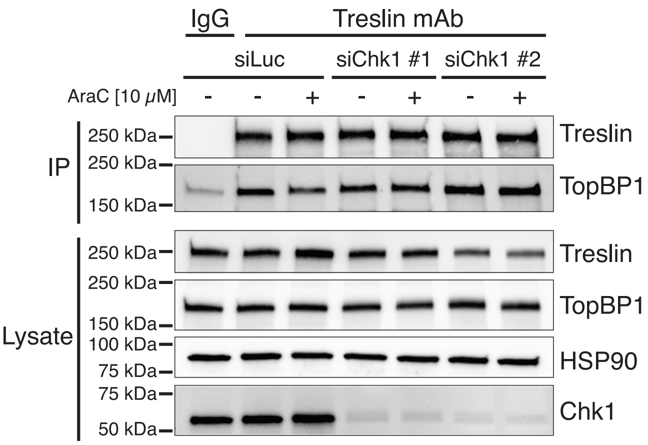

Supporting Figure S2. U2OS cells were transfected with siRNA targeting luciferase (siLuc, negative control) or two different Chk1 siRNAs. After 48 hours, cells were treated for 2 hours with AraC and lysed. Lysates were immunoprecipitated (IP) with mouse immunoglobulin G (IgG) control antibody or Treslin monoclonal antibody (mAb) pre-bound to protein G agarose beads. Immunoprecipitates and lysates were immunoblotted for the indicated antigens.

Supporting Figure S3

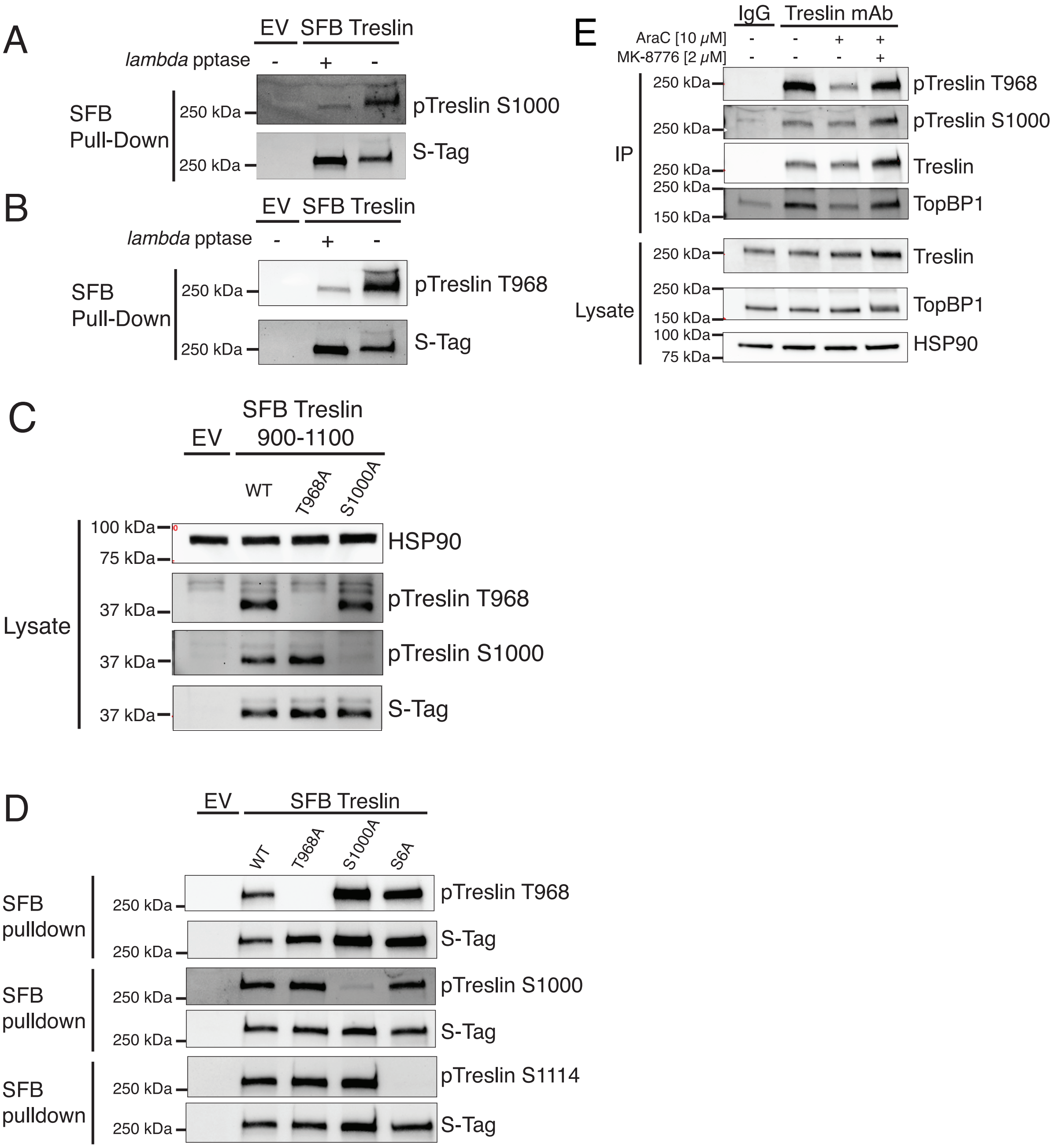

Supporting Figure S3

Supporting Figure S3 A-B. K562 cells were lysed 24 hours after transfection with pIRES SFB Treslin WT, and lysates were incubated with streptavidin agarose beads. After washing, beads were incubated in phosphatase buffer for 30 minutes at 30 °C with or without lambda phosphatase (pptase) as indicated. SFB Treslin was eluted from beads, and membranes were immunoblotted with anti-phospho-S1000 Treslin (pTreslin S1000) (A) and anti-phospho-T968 treslin (pTreslin T968) (B) antibodies. **C.** Lysates from K562 cells transfected with pIRES SFB Treslin 900-1100 WT or the indicated mutants were immunoblotted for Treslin phospho-T968 and phospho-S1000 Treslin. **D.** pIRES SFB Treslin WT, T968A, S1000A, and S6A were overexpressed in K562 cells. 24 hours later, cells were lysed, and lysates were incubated with streptavidin agarose beads for pulldown of the SFB-tagged protein. Pulldowns were immunoblotted for Treslin phosphorylation sites: phospho-T968 (pTreslin T968), phospho-S1000 (pTreslin S1000), and phospho-S1114 (pTreslin S1114). **E.** U2OS cells were treated for 4 hours with the indicated agents prior to lysis. Lysates were incubated with protein G agarose beads pre-bound with mouse IgG monoclonal control antibody or Treslin monoclonal antibody (mAb). Immunoprecipitates (IP) and lysates were immunoblotted for the indicated antigens.

Supporting Figure S4

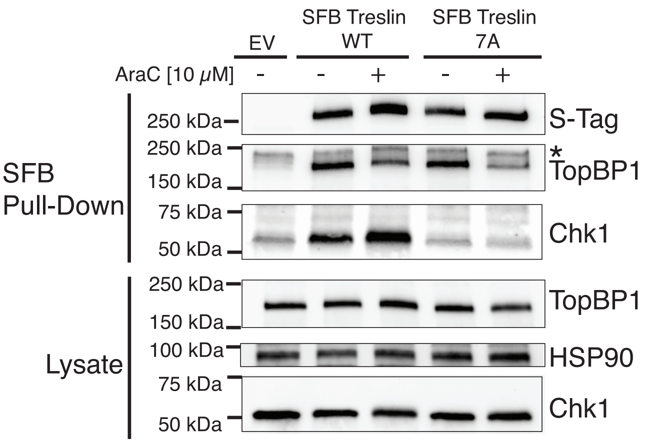

Supporting Figure S4. K562 cells were transfected with pIRES SFB Treslin WT or pIRES SFB Treslin 7A. 24 hours later, cells were treated for 4 hours with the indicated agent prior to lysis. Lysates were incubated with streptavidin agarose beads. SFB Treslin and interacting proteins were eluted from beads, run on a gel, and blotted for the indicated antigens.

Supporting Figure S5

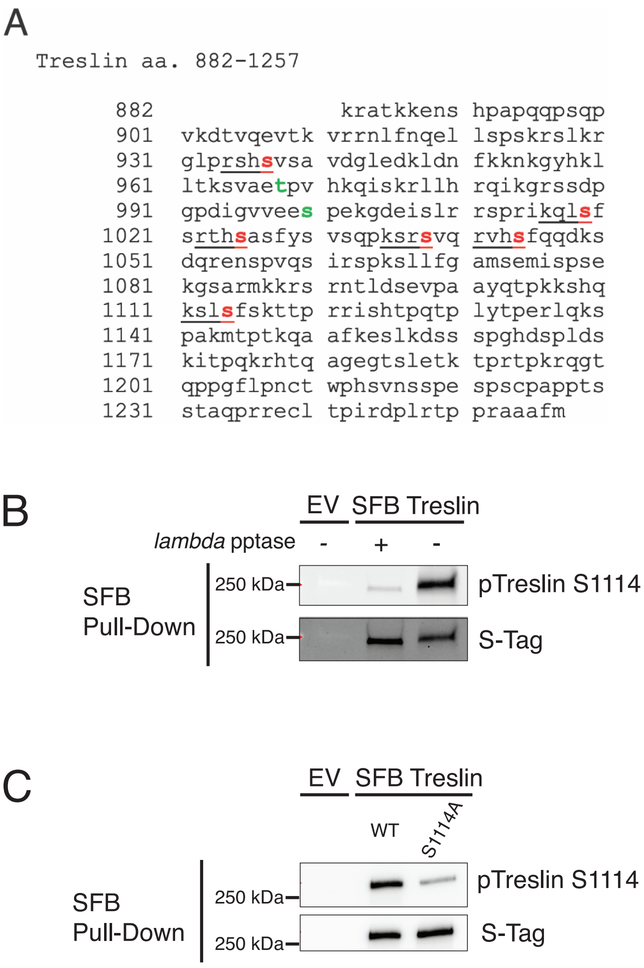

Supporting Figure S5. A. Sequence of Treslin from amino acids 882-1257 with T968 and S1000 indicated in green and with six Chk1 consensus sites (S937, S1019, S1025, S1038, S1044, S1114) that are mutated to alanines in Treslin S6A, indicated in red. **B.** K562 cells transfected with pIRES SFB Treslin WT were lysed and lysates were incubated with streptavidin agarose beads. After washing, beads were incubated in phosphatase buffer for 30 minutes at 30 °C with or without lambda phosphatase as indicated. Fragments were eluted from beads, run on a gel, and blotted with pTreslin S1114 antibody. **C.** pIRES SFB Treslin WT or pIRES SFB Treslin S1114 were overexpressed in K562 cells. Cells were lysed and lysates were incubated with streptavidin agarose beads for pulldown of the overexpressed protein. Pulldowns were immunoblotted for Treslin phospho-S1114 (pTreslin S1114).

Supporting Figure S6

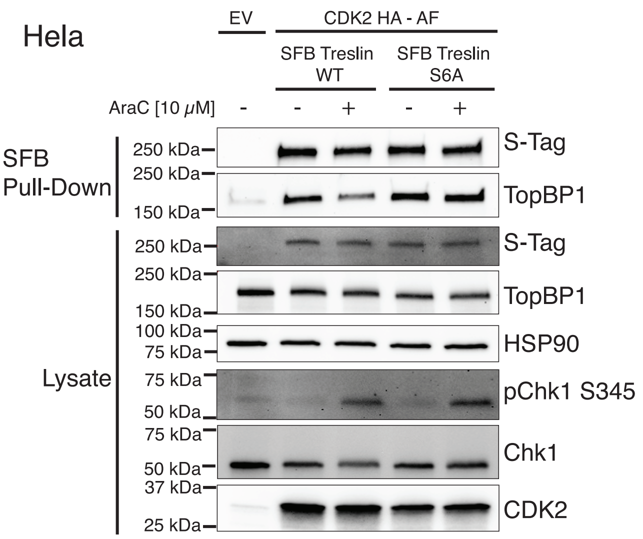

Supporting Figure S6. HeLa cells were co-transfected with either empty vector, pIRES SFB Treslin WT, or pIRES SFB Treslin S6A along with pCMV CDK2 HA AF, as indicated. 48 hours later, cells were treated with AraC for 2 hours and collected for lysis. Lysates were incubated with streptavidin agarose beads to pulldown SFB-tagged proteins. Lysates and bead-bound proteins were immunoblotted for the indicated antigens.

Supporting Figure S7

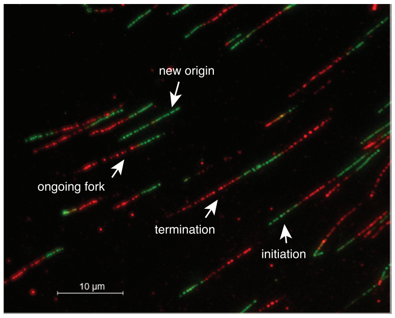

Supporting Figure S7

Example image of DNA Fiber Labeling events:

ongoing fork: red-green

initiation: green-red-green

termination: red only, or red-green-red

new origin: green only

1. Hackbarth, J. S., Lee, S. H., Meng, X. W., Vroman, B. T., Kaufmann, S. H., and Karnitz, L. M. (2004) S-peptide epitope tagging for protein purification, expression monitoring, and localization in mammalian cells. *BioTechniques* **37**, 835-839
